# Progress in the study of aging marker criteria in human populations

**DOI:** 10.3389/fpubh.2024.1305303

**Published:** 2024-01-24

**Authors:** Yan He, Zhe Li, Yue Niu, Yuting Duan, Qian Wang, Xiaomin Liu, Zheyi Dong, Ying Zheng, Yizhi Chen, Yong Wang, Delong Zhao, Xuefeng Sun, Guangyan Cai, Zhe Feng, Weiguang Zhang, Xiangmei Chen

**Affiliations:** ^1^Chengdu University of Traditional Chinese Medicine, Chengdu, China; ^2^Department of Nephrology, First Medical Center of Chinese PLA General Hospital, National Key Laboratory of Kidney Diseases, National Clinical Research Center for Kidney Diseases, Beijing Key Laboratory of Kidney Diseases Research, Beijing, China; ^3^The First Affiliated Hospital, College of Clinical Medicine of Henan University of Science and Technology, Luoyang, China; ^4^Department of Nephrology, Hainan Hospital of Chinese PLA General Hospital, Hainan Province Academician Team Innovation Center, Sanya, China

**Keywords:** marker, aging, aging markers, human, biological age, chronological age

## Abstract

The use of human aging markers, which are physiological, biochemical and molecular indicators of structural or functional degeneration associated with aging, is the fundamental basis of individualized aging assessments. Identifying methods for selecting markers has become a primary and vital aspect of aging research. However, there is no clear consensus or uniform principle on the criteria for screening aging markers. Therefore, we combine previous research from our center and summarize the criteria for screening aging markers in previous population studies, which are discussed in three aspects: functional perspective, operational implementation perspective and methodological perspective. Finally, an evaluation framework has been established, and the criteria are categorized into three levels based on their importance, which can help assess the extent to which a candidate biomarker may be feasible, valid, and useful for a specific use context.

## Introduction

Currently, countries worldwide face a difficult aging situation, and the intensification of aging will bring a heavy medical burden (1–4) and a social burden (5) to the world. Aging is a gradual, progressive process, involving the accumulation of molecular, cellular, and organ damage, resulting in decreased physical and cognitive functions and increased susceptibility to diseases (6). Therefore, the WHO, China, and some other countries have also changed the goal of aging research from “active aging” to “healthy aging.” However, to achieve healthy aging, it is necessary to correctly assess the functional status of the organs of aging individuals. The functional status of different individuals with the same chronological age (CA) may be different, and CA may be insufficient in assessing the functional situation of aging individuals. Previous researchers used aging markers as an alternative indicator of CA and also used a personalized evaluation model combining multiple markers, such as biological age (BA) (7–10). Aging markers are the basis for individualized evaluation of aging, and the selection of aging markers is crucial for the individual assessment of aging function. There are similarities and differences in the screening criteria proposed by previous studies (10–12). In this review, we combine previous research from our center (10, 13–17), summarize and discuss the current views on screening criteria for aging markers, related problems of existing criteria, and solutions, and compare their use. Finally, an assessment framework has been established, and the criteria are categorized into three levels based on their importance.

## Screening criteria for aging markers

### Search methods

We searched PubMed, MEDLINE, and Web of Science databases for literature between 1 January 1962 and 31 August 2023. The search key terms were “aging markers,” “markers of aging,” “biomarkers of aging,” or “biological age” and were limited to full-text articles published in English. The search initially identified 5,014 potentially relevant articles, excluding those not published in English and those not available in full text. Duplicate and irrelevant studies are excluded by carefully reviewing titles, abstracts, and full texts. Finally, a total of 85 studies on the construction of population biological age and reviews on the criteria of population aging markers were included.

**Figure 1:**
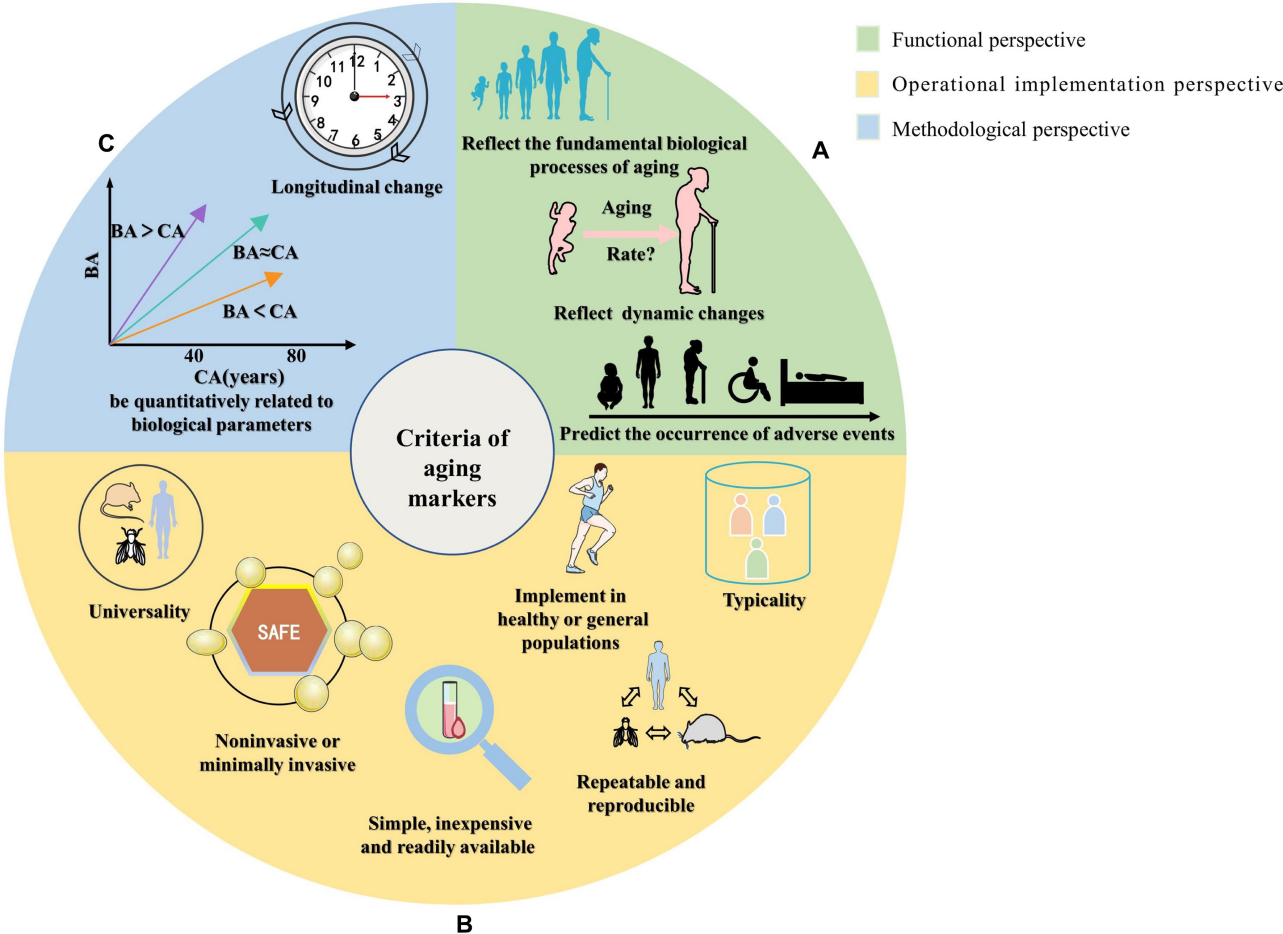
Schematic diagram of the classification of screening criteria for aging markers. The summarized screening criteria for aging markers are divided into three aspects: **(A)** Functional perspective, reflecting the fundamental biological processes of aging; reflecting dynamic changes in a short period (predicting the aging rate); predicting the occurrence of adverse events. **(B)** Operational implementation perspective, which involves measures that are noninvasive or minimally invasive; simple, inexpensive, and easily accessible; repeatable and reproducible; can be measured in multiple species; should represent the function of an organ or system; and can be implemented in healthy or general populations to avoid disease interference. **(C)** Methodological perspective that involves quantitative correlation with biological parameters and verification of longitudinal changes with age that is consistent with cross-sectional relationships.

This review summarizes the criteria for screening aging markers mentioned in the literature, as shown in Supplementary Table 1. We classified the screening criteria into three aspects: (i) Functional perspective: reflecting the fundamental biological processes of aging; reflecting dynamic changes in a short time (predicting the aging rate); predicting the occurrence of adverse events. (ii) Operational implementation perspective: noninvasive or minimally invasive; simple, inexpensive, and easily accessible; repeatable and reproducible; can be measured in multiple species; should represent the function of an organ or system; implementation in healthy populations to avoid disease interference. (iii) Methodological perspective: quantitative correlation with biological parameters; verification of longitudinal change with age that is consistent with cross-sectional relationships, as shown in Table 1 and Figure 1.

**Table 1:** Classification of criteria for screening aging markers.

Categories	Criteria	Contents of the Criteria
Functional perspective	Biological: Reflect the basic biological processes of aging	Cover a range of physical functions Can monitor the processes associated with aging characteristics Can reflect the aging changes consistent with aging
	Dynamics: Reflect dynamic changes in a short time	Can reflect the rate of aging over a short period
	Predictability: Predict the occurrence of adverse events	Can predict life expectancy and healthy life span Can predict individual mortality Can assess age-related disease and mortality Can predict the endpoint events associated with aging Can predict the risk associated with endpoint events Sensitive to the early signs of aging Can identify high-risk populations before disease occurrence
Operational implementation perspective	Noninvasive/minimally invasive	Safe and harmless manner without increasing the burden on the patients No reduction in life expectancy, no change in subsequent outcomes
	Implementability: Simple, inexpensive and readily available	Reliable and easy to observe Easy to calculate and measure Universal, commonly used in clinical practice
	Stability: Repeatability and reproducibility	Highly reproducible Stable indicators
	Universality: Measurable in multiple species	Measurable across species Validated in model animal studies and then in human experiments
	Typicality: Representative of the function of an organ/system	Markers are representative Markers are independent of each other and not redundant
	Implementing populations: Implemented in healthy or general populations	Aging markers should be obtained in disease-free, healthy populations Aging process is not affected by disease
Methodological perspective	Quantifiable: Quantitative correlation with biological parameters	Independent, high, quantitative, cross-sectional correlation with CA
	Verifiable	Significant longitudinal variation with age Consistent with cross-sectional relationship

The criteria for screening aging markers and their specific contents are divided into three categories.

## Functional perspective

### Biological: aging markers should reflect the fundamental biological processes of aging

CA cannot dynamically or accurately evaluate the functional status of aging individuals. Therefore, alternative indicators such as aging markers (18, 19) are needed to better assess aging. The most basic and vital requirements for selected aging markers are that they can represent or reflect the fundamental biological process, be correlated with aging characteristics, reflect aging-consistent aging change (20, 21), reflect better aging-related biological and functional outcomes than CA (22–26), and cover a range of physical functions. Then, several aging markers can be combined to construct a functional age to better reflect aging individuals’ functional status. In previous studies at our center, various biological age models were constructed by combining different aging biomarkers and using different methods. Zhang et al. (10, 17) constructed a biological age equation using several aging markers. Li et al. (16) used nine markers and eight methods to assess BA in the Chinese Han population.

### Dynamics: aging markers reflect dynamic changes in a short time

Aging markers can predict the rate of senescence (10, 20, 21, 27, 28) in a relatively short time and can monitor the aging process and identify individual differences in this process. The most accurate way to calculate aging rates is to use a longitudinal follow-up over a long time or to track and observe the whole process of a person from birth to aging and then to death. This is the most accurate way to calculate the aging rate. However, from the perspective of implementation, long-term longitudinal follow-up is more difficult to implement, and indicators are needed to judge the aging status of an individual at the time of survival analysis. Therefore, aging markers must be able to reflect dynamic changes in a short time.

### Predictability: aging markers can predict the occurrence of adverse events

Aging markers can predict disease occurrence and individual organ/system functional decline (29–35) and even predict the lifespan and healthy lifespan (36–38). BA is a better predictor of life expectancy than CA. Aging markers can also be used to predict individual mortality, assess and predict age-related disease and mortality (26, 37, 39–43), predict aging-related endpoint events such as functional decline, quality of life, and survival rate, predict the risk associated with endpoint events, assess the magnitude of risk, provide information according to the health-related results of the assessment, and enable early intervention to improve healthy survival (22, 44–48). These factors indicate the tremendous importance of aging markers. Good aging marker can identify the risks of early aging and is sensitive to whether a person is at high risk of diseases or in the early stages of disease in a specific population. It can screen out the high-risk groups of illness before the occurrence of diseases and provide early warning of conditions before treatment, which can help with disease diagnosis and the provision of timely and appropriate guidance, intervention, and treatment (24, 49).

## Operational implementation perspective

### Noninvasive: aging markers should be noninvasive or minimally invasive

The procedure and method of obtaining aging markers should be noninvasive or minimally invasive (21, 41, 50). At the same time, the measurement method must not reduce the life expectancy of the model organisms and humans or alter any subsequent results related to age-sensitive testing and must be able to be repeated in a safe, harmless manner without increasing the burden on the patients (20, 51–53). The detection of aging markers should be easy. However, some indicators require invasive manipulation (e.g., biopsy sampling). Although some markers can be measured during planned surgical or pathological testing (although the results are less reproducible), obtaining markers invasively during aging studies in healthy populations may not be easy.

Generally, there are two types of aging markers: those detected by obtaining tissue or biological samples and those detected by external instruments or by some physiological indicators of the organism. Some samples, such as blood markers, are minimally invasive, while others are noninvasive, such as markers detected in saliva and urine. In addition, some imaging or instrumental tests, such as ultrasound, handgrip strength, pulse wave velocity, and blood pressure, are non-invasive. Due to ethical requirements, applicability and feasibility considerations, and the fact that biological age is usually constructed using a healthy or general population, it is impractical to perform major invasive procedures such as tissue extraction, and therefore, minimally invasive or non-invasive procedures are very important.

### Implementability: aging markers should be simple, inexpensive, and readily available

Markers of aging are easy to observe and reliable; they are also easy to calculate and measure (33, 46, 54), and from a clinical or practical point of view, aging markers should be simple, universal, frequently used in clinical practice (33, 55, 56), inexpensive (21, 23, 33, 50, 55, 57), rapid and methodologically relatively homogeneous, readily available (31, 33, 38, 44, 45, 54, 56), and measured with realistic, standardized results (21, 24, 42). However, many new detection methods and indicators exist, such as genetic indicators and magnetic resonance imaging (PET, MRI), artificial intelligence, machine learning, and multiomics studies. Implementing these assays and indicators may require high costs, professional equipment, technicians, and many samples. Nevertheless, they also have multiple future applications and benefits worth exploring and studying.

### Stability: aging markers should have repeatability and reproducibility

Markers of aging should be relatively stable so that their measurements are repeatable and reproducible (49, 53, 58, 59). Several studies have shown that aging markers follow a highly reproducible pattern during aging and can be obtained by repeated measurements, showing robust stability. At the same time, it needs to be repeated without affecting the health or longevity of the model organism and humans (41, 45, 51, 60), although some unstable indicators may accurately reflect changes in the aging body. For example, the glomerular filtration rate is positively correlated with CA (r = 0.33). However, volatile markers cannot be directly detected and therefore cannot be used as aging markers.

### Universality: aging markers can be measured in multiple species

Aging markers can identify individuals with the same CA but different functional states and thus assess aging status (14). Individual differences in species suggest that aging processes and environmental conditions that control or influence lifespan differ from those in disease states (61). However, many researchers believe that common aging markers should also be involved in the aging process in many rodents. Aging markers are commonly used in multiple systems or organs and can be measured in a variety of species or across species (54, 62) and obtain stable results (22, 63); they generally require experiments in laboratory organisms (e.g., rats) before being validated in humans. They need to produce effects in humans and laboratory animals (21, 53).

### Typicality: aging markers should represent an organ/system function

Aging markers should not be redundant with each other and should not have too much correlation. They should have separate sensitivity and specificity characteristics and independent functions so that they can be used as indicators of predictive ability at the individual level, reflect individual physiological states, contribute to disease diagnosis (46, 48), and reveal the mechanisms of disease occurrence and homeostatic changes in the personal life cycle (23, 46, 64). They should provide the most robust representation of the aging process and represent an organ or system without significant redundancy with other selected variables (33, 65). For example, systolic blood pressure, pulse pressure, and the pulse pressure index all meet the criteria for markers of aging; nevertheless, they are also highly correlated (e.g., one variable can be used to determine the other two). Thus, there is redundant information for use in the construction of biological age. Therefore, if methods such as traditional principal component analysis are applied, and 6–10 markers are ultimately chosen to construct biological age, it is recommended that one of these indicators be selected to reflect blood pressure status, and this principle is also applicable to other organs/systems; If the new artificial intelligence methods are applied to construct biological age, more markers may be selected to reflect the same information, because different markers can reflect different information even if there is a high correlation. Of course, it is paradoxical that fewer markers are chosen for better implementation while more markers are selected for improved accuracy.

### Non-pathological: implemented in healthy or general populations to avoid disease interference

Although some studies have shown that disease and aging are unrelated processes, some scholars have argued that they are generally accepted as independent but interrelated processes (66). However, many studies have included diseased individuals (or failed to account for the effects of individual disease). They have screened out markers that are not associated with aging but may be associated with the disease. Therefore, the obtained markers cannot be considered authentic aging markers. Ideally, aging markers screened in healthy populations exclude the influence of disease on aging. They can better reflect the alterations and process changes brought by aging, which is essential for maintaining healthy aging. Therefore, aging marker screening requires disease-free healthy people (35, 61, 67–69), and the basic biological process of aging is not affected by the disease process.

## Methodological perspective

### Quantifiable: aging markers should be quantitatively related to biological parameters

One of the main criteria for selecting aging markers is that they should be quantitative (29, 67). Klemera and Doubal proposed using mathematical modeling to elaborate the association between CA and BA (12, 70), which have a high, independent, and quantitative correlation (37, 49, 59, 71, 72). Aging markers can quantitatively assess the aging process and the degree of delayed aging, potentially assess the risk of aging-related diseases and provide direction for personalized interventions (52, 64, 73). Aging markers have a quantitative correlation with biological parameters and subject age and a significant cross-sectional correlation with age. Researchers have suggested that a potential marker’s quantitative relationship with CA can be used as one of the criteria for screening aging markers (62, 65, 67, 74–77). Therefore, aging markers can be selected as a CA substitute for assessing the extent of physical aging.

### Verifiable: there is a significant longitudinal change in aging markers with age, consistent with the cross-sectional relationship

Although cross-sectional studies may correlate potential markers with CA, this result is insufficient to identify aging markers. Significant longitudinal changes with age are consistent with the results of cross-sectional studies, which is a significantly efficient validation process. Cross-sectional designs can only show differences between specific time points and age groups; they can neither be used to conclude that changes occur as a result of aging phenomena nor be used to explain why this pattern varies by age group. Therefore, it is necessary to further confirm the marker with longitudinal studies at the same level as cross-sectional studies (10, 11, 19, 62, 65, 74, 75, 77). Studies have shown that performing longitudinal follow-up and in-depth multivariate analysis is necessary to help identify markers of aging, evaluate the stability of the marker, reflect the aging process, and measure the rate of aging (40, 58). The changes in aging markers over time have been validated longitudinally, and there are different patterns of aging that can be used to help monitor and intervene in the changing process of aging (78). Therefore, a longitudinal design can more thoroughly explain the changes in aging over time. If implemented in the construction of biological age, statistical internal cross-validation, external validation, and longitudinal validation play a critical role in enhancing the accuracy of biological age.

## Problems associated with the criteria for screening aging marker and their solutions

Alex Comfort first proposed that age-related biological changes could be measured in 1969, and changes in aging can be quantified by identifying and measuring aging biomarkers (79, 80). From 1988 to 1998, the National Institute on Aging launched a 10-year initiative to encourage research on biomarkers of aging. Although researchers have explored many possible biomarkers as candidates and contributed to the knowledge base of aging, no specific biomarkers have been successfully identified and validated. Due to the complexity of the aging process, especially in humans, there is no single aging marker in an absolute sense, and no single marker can predict the rate of aging. The previously proposed marker is conceptually similar to functional age or BA, consisting of several markers. No single marker can satisfy the need for BA or other CA alternatives.

Although researchers have proposed several consensus criteria for selecting aging markers, there are still many issues to be resolved, for instance, the selection of baseline and study populations and whether aging markers are affected by the disease. This section discusses these issues and tentatively proposes solutions to them.

### What is the relationship between disease and aging?

Ideally, aging markers reflect the healthy aging process in individuals not influenced by disease, but there is no ideal marker, nor are there markers in the literature that are pure biomarkers of aging; for example, cystatin C is a marker of aging and kidney disease. There has been a debate about aging as a cause or a consequence of disease.

Disease and aging may have an interactive and causal relationship. Markers of aging may be markers of disease, but markers of disease may not be markers of aging. On the one hand, the disease process will accelerate the aging process (81); on the other hand, aging increases the susceptibility to disease and thus accompanies many chronic diseases. The disease process has many mutual processes with normal aging; the aging process accompanies the disease process, which may accelerate the normal aging process.

### Are there nodes in the aging process? Is the rate of aging constant?

Some researchers have suggested that the aging rate is not uniform and that nodes exist (82), i.e., points at which the aging rate undergoes acceleration or deceleration. We consider the rate of aging to be homogeneous for the whole population. However, for individuals, there could also be certain nodes that lead to a changed aging rate, such as disease or lifestyle changes.

The study’s sample size is small compared to the studies proposing the existence of nodes, which may lead to bias in the results after grouping. Adequate sample size and longitudinal follow-up are needed to verify this conclusion. Theoretically, aging is a uniform process, and the rate of aging does not change in a short time; however, some factors may contribute to changes in aging rates: ① relatively slow aging after the age of 80 or 90 in certain special populations, such as centenarians or the children of centenarians; ② environmental factors and behavioral changes (obesity, smoking, drug use); ③ genetic differences and chronic diseases that accelerate the aging process; and ④ unpredictable accidents (24).

### Can aging markers predict life expectancy?

Some researchers believe that aging markers can predict individual longevity or mortality. If the aging process is purely healthy, it is possible to predict the incidence of disease, individual lifespan, and mortality. However, in reality, on the one hand, we need longitudinal studies lasting until death to derive aging rates, which is a very long time in human studies; on the other hand, lifespan becomes affected by too many uncontrollable and unpredictable factors that arise, such as sudden malignant diseases (tumors) and acute diseases (acute cardiovascular diseases) (83). In addition, there are significant genetic factors involved, and lifespan is determined by approximately 20%–50% of genetic factors, but the most commonly used biomarkers have no genetic indicators, and their accuracy in predicting life expectancy is questionable.

Therefore, aging markers can predict the duration of the healthy period and the occurrence of diseases or suggest related complications. However, if aging markers are to be used to predict lifespan and mortality, more prerequisites are needed: (i) the aging process is a healthy aging process, excluding the influence of disease factors; and (ii) the interference of unpredictable diseases, accidents, psychological and environmental factors are excluded (22).

### Are aging markers universal or specific?

Some researchers have proposed that markers should be universal, meaning they are required to be valid in humans and model organisms. Experiments are performed in model organism models (e.g., caloric restriction, genetic genes, etc.) and then validated in humans; certain aging characteristics are found in humans and need to be experimentally validated in model organisms due to the requirements of human ethics. Although general indicators can be applied to various species, there are also genetic differences between species; psychological and social factors may significantly affect human aging, which cannot be studied in some mammalian or lower animal models. Therefore, aging markers must be applied to all categories and species, which is not easy to achieve. Aging markers in humans and different species of model organisms may be very different, and model organisms can be applied appropriately but not mechanistically.

### Should screening be done according to quantitative criteria?

Many researchers have noted that aging markers should be measurable and quantitative indicators that change with age. However, although traditional thinking holds that only continuous quantitative variables can be used as aging markers, with advances in aging markers and technology, some semiquantitative and qualitative indicators have been included as candidate aging markers, including certain genes (84, 85), telomere length (9, 86), DNA methylation (87, 88), copy number variables (89), and multiomics indicators.

Aging markers are quantitatively, highly and independently correlated with CA, and such correlations are initially identified with mostly linear and multiple logistic regression analyses. Some nonlinear statistical methods have been applied; initially, there was no correlation between aging markers and CA, but after processing, they became correlated again or were screened by machine learning. Other methods may not have linear correlation, which may indicate a more complex relationship, but does not mean that such markers cannot become aging markers. Therefore, we should decide whether to follow the screening criteria of quantitative correlation analysis according to the actual method employed.

### How should the study population be selected?

Aging population studies, including centenarians and their children, long-lived people, twins, etc., have been studied in aging marker research. Healthy populations are best for aging mechanisms or aging marker studies, which have the advantage of avoiding interference from disease. Longevity is usually closely related to environmental and genetic factors, and results obtained in specific regions and environments may not apply to the general population; moreover, the odds of chronic comorbidities or carrying chronic diseases are higher in long-lived people.

Therefore, how can the population be selected in aging marker research? Is it that you cannot select anyone other than those in healthy populations? Not truly. Choosing healthy and relatively healthy people for a study can effectively exclude confounding factors such as disease; if the aging acceleration model disease population or the progeria population is chosen, the outcomes and endpoint events can be seen more quickly, each with its advantages and disadvantages.

### Are there other options for selecting benchmark markers apart from their correlation with CA?

The selection of aging markers is generally based on a benchmark marker, and the most commonly used benchmark marker is *CA.* Because of the limitations of CA itself, alternative indicators need to be selected for the evaluation of aging. Using CA as a benchmark for determining new alternative markers is relatively contradictory. Previous studies have also explored different methods for screening biomarkers. In addition to statistical correlation, other noncorrelations, exponential correlations, power correlations and even some noncorrelations that are tried to be applied in the aging process can be used as aging markers (12, 90). In addition to applying principal component or multivariate linearity after traditional dimensionality reduction, new machine learning methods may provide new ideas, such as using importance ranking (91). In addition to traditional CA, dual or multiple criteria, such as CA combined with telomere length or handgrip strength, maybe more accurate in selecting aging markers (14, 15). Whether alternative benchmark markers can adequately reflect the dynamic changes that occur during the aging process and replace CA or be combined with CA as a benchmark marker for screening aging markers requires further scientific research. New ways of selecting benchmarks are also being further explored.

### What statistical methods should be applied?

Several different methods are used when determining correlations among the selected markers, including multiple linear regression, Klemera and Doubal (KD) methods and principal component analysis, to select different markers in the same population (22), with categorical, binary or continuous variables as input. Candidate markers can be used as aging markers if the correlation coefficient (r) is greater than a set threshold. For the correlation coefficient threshold, there is disagreement. For example, correlation coefficients have been set at 0.40, 0.25 (92), or even 0.15 (27) as the thresholds for aging marker screening. The larger the correlation coefficient is, the more valid the selected aging markers should be. Previous studies of aging markers greater than 0.4, including grip strength, simple reaction time (RT), visual conditioning, visual acuity, auditory acuity (4,000 Hz), digit symbols, systolic blood pressure, diastolic blood pressure, spirometry, and expiratory volume with force (93), can be used as aging markers.

In recent years, machine learning and artificial intelligence algorithms have also been used to identify aging markers that significantly impact biological age (91). However, the new methods are not always better, as new technologies and methods such as machine learning require a large sample size as a basis. Too little data may be overfitted, and being a “black box” process, the specific learning process is unknown and the results produced are uncontrollable. Meanwhile, the traditional methods also have different limitations. Therefore, it is necessary to choose a suitable method according to the actual situation of the sample size.

### Can exogenous markers be applied?

Based on advances in technology, updates in perception, and advances in statistics, aging markers have undergone a progression from traditional physiological markers to biomarkers to molecular and cellular markers and the latest fourth generation of histological markers (43). Not only are traditional internal physiological biomarkers reflecting biological characteristics (e.g., blood, urine) valued, but in addition, with advances in image recognition technology, some photographs such as retinal photographs, three-dimensional (3D) facial data, and deep learning of structural neuroimaging can also be used as markers or even as a functional age to reflect individual health status (73, 94, 95). However, it has become clear that external factors (e.g., diet, environment, temperature, environmental radiation, exercise, lifestyle, and psychological and socioeconomic status) have also influenced aging and are thus receiving attention from researchers.

## Summary and prospects

CA is inadequate in assessing the aging process and aging rate of individuals (12, 96, 97). Thus, alternative indicators are needed to select appropriate aging markers, accurately evaluate and detect high-risk individuals, and intervene early to help improve the quality of survival and prolong the life span and healthy lifespan of patients (12, 98, 99). Therefore, it is critical to screen for appropriate and prospective markers.

**Figure 2:**
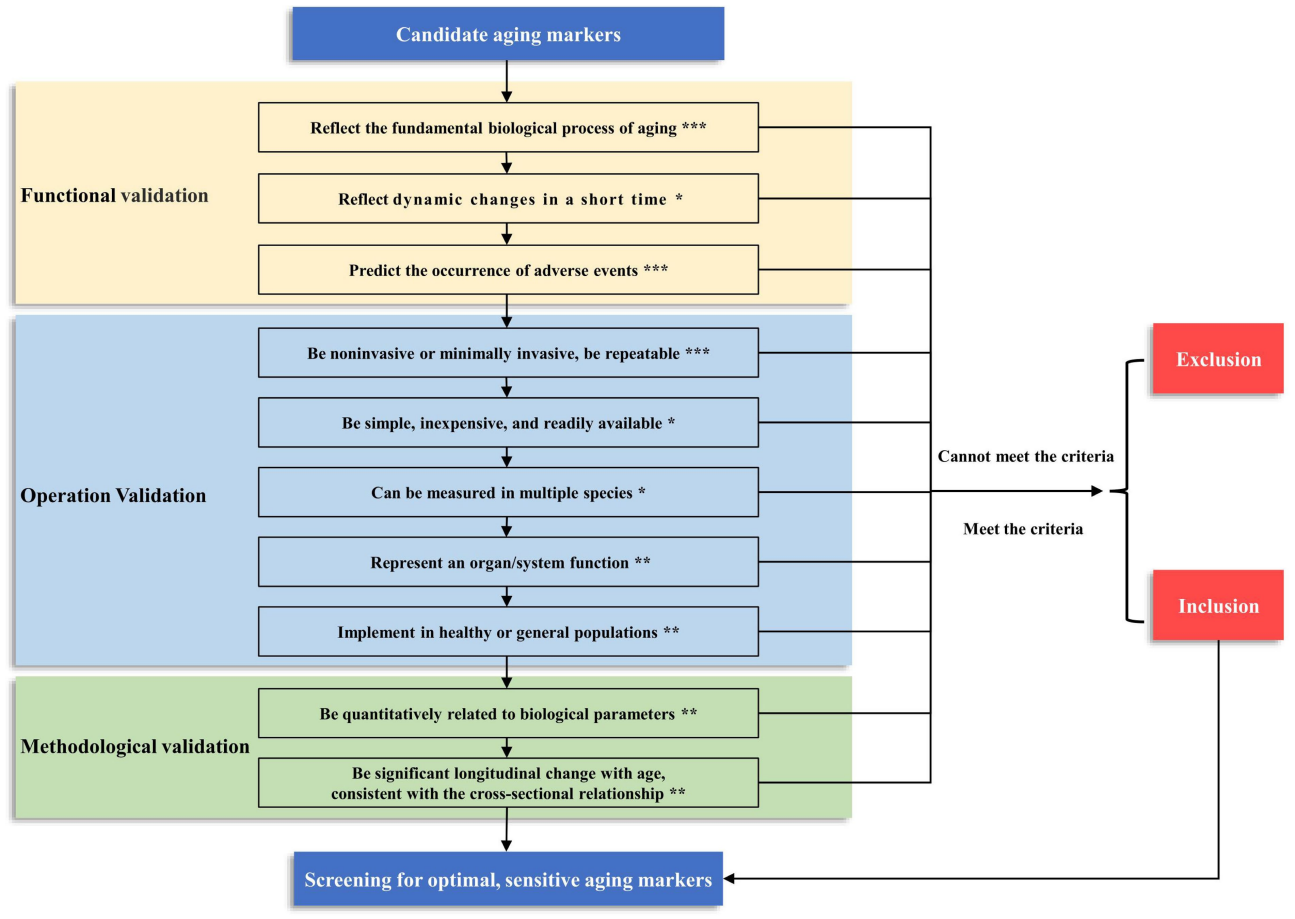
The framework and grading of criteria for screening aging markers. We have categorized the criteria into three levels based on their importance: ***Level 1, the criteria that must be met; **Level 2, the criteria recommended to meet; *Level 3, the criteria that may be met. When constructing the biological age of a population, the three criteria of level 1 must be met before they can be included as candidate markers. In addition, the more criteria that are satisfied at level 2 and level 3, the better. This screening process can yield optimal and sensitive aging markers.

**Figure 3:**
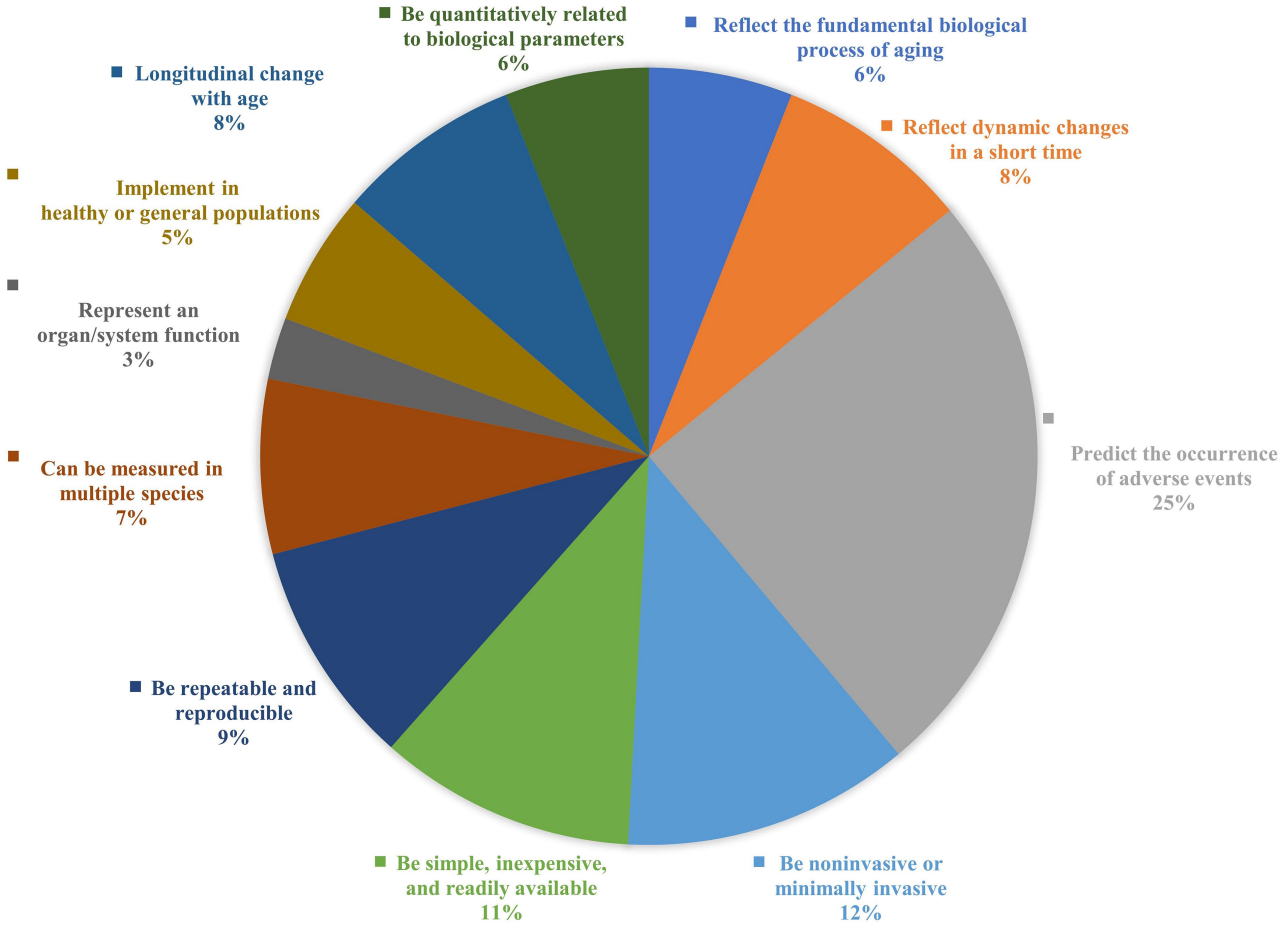
Comparisons on the frequency of occurrence of the criteria in previous studies. A summary of the frequency of occurrence of each criterion is based on the studies summarized in Supplementary Table 1, ranked from high to low in terms of frequency of use: predict the occurrence of adverse events (25%); be noninvasive or minimally invasive (12%); be simple, inexpensive and readily available (11%); be repeatable and reproducible (9%); reflect dynamic changes in a short time (8%); longitudinal changes with age (8%); can be measured in multiple species (7%); be quantitatively related to biological parameters (6%); reflect the fundamental biological processes of aging (6%); implemented in healthy or general populations (5%); represent an organ/system function (3%).

To better guide screening aging biomarkers in future studies, we summarize the criteria for screening aging markers in previous studies (Supplementary Table 1). A framework for aging marker characterization and assessment has been developed (Table 1, Figures 1, 2). Comparisons are also made based on the frequency of occurrence of the criteria in previous studies (Figure 3). The top four criteria are predicting the life expectancy and the occurrence of adverse events; noninvasive or minimally invasive; simple, inexpensive, readily available; and repeatable, reproducible. These criteria are generally consistent with the criteria mentioned in previous studies (11, 69, 94) and criteria recently proposed by the Biomarkers of Aging Consortium (98, 99).

We combine previous research from our center (10, 13–17) and categorize the criteria into three levels based on their importance. Level 1, the following criteria must be met: ① aging markers should reflect the fundamental biological process of aging; ② aging markers can predict the occurrence of adverse events; and ③ aging markers should be noninvasive or minimally invasive and be repeatable. At Level 2, the following criteria are recommended: ① aging markers should represent organ/system function; ② aging markers should be implemented in healthy or general populations; ③ aging markers should be quantitatively related to biological parameters, and ④ aging markers should have significant longitudinal changes with age, consistent with the cross-sectional relationship. At Level 3, the following criterion may be met: ① aging markers reflect dynamic changes in a short time; ② aging markers can be measured in multiple species; and ③ aging markers should be simple, inexpensive, readily available. Based on our previous research, we suggest that three criteria of level 1 must be met when constructing the biological age of a population before it can be used as a candidate marker (Figure 2). However, a single biomarker makes it difficult to identify individuals at risk who can meet the diagnostic criteria. Therefore, a single optimal biomarker is not recommended, and a reliable combination of biomarkers is needed. The combination of biomarkers that meet these criteria can provide a more accurate assessment of aging, which can multidimensionally predict biological age and the risk of certain diseases in specific organs, which can help translate these findings into clinical practice (98, 99).

We believe that reflecting the fundamental processes of aging, being quantitatively related to biological parameters and implemented in healthy or general populations to avoid interference from disease factors but with results that can be extended to diseased populations, being able to predict the occurrence of adverse outcomes, and being able to reflect the function of vital organs or systems are promising screening criteria. However, some criteria have yet to receive sufficient attention, such as reflecting the fundamental biological processes of aging, being quantitatively related to biological parameters, being implemented in healthy or general populations, and being representative of the function of an organ/system. The quantitative correlation may have changed because of the application of new statistical methods, and several other methods should be emphasized. Some studies have proposed that some criteria for screening biomarkers are derived from or applicable to basic research (99, 100). However, due to the specificity of the population, these criteria may not be suitable for screening biomarkers in the population from the point of ethics and human safety. Therefore, we propose that the three criteria at level 1 must be met when constructing the biological age in the population. In addition, the more criteria that are satisfied at level 2 and level 3, the better. The accuracy, reliability, and safety of the screened markers will be significantly improved.

In assessing aging and constructing biological age, the aging markers depend on three aspects: conceptual, technical and methodological advances. Conceptual advances indicate the development of the concept of aging markers is no longer being limited to “biological factors” reflecting the aging process but rather to factors that can influence the biological outlook (97, 100). Technological advances indicate that aging markers have evolved from traditional physiological and biological markers reflecting biological characteristics to molecular, cellular, and even omics markers, as well as to psychological, economic, sociological, environmental, and genetic indicators. The development of noninvasive and minimally invasive imaging technologies, as well as high-throughput sequencing, single cell sequencing, and transcriptomics has also enriched the database of aging candidate markers (73, 94, 95, 98). The progress of methods indicate that the screening of aging markers may involve more than superficial linear relationships, and with the development of mathematical models, qualitative and semiquantitative markers can also be used as potential candidate aging markers. New statistical methods, especially the application of machine learning, and markers identified by nonlinear correlation techniques are likewise more widely used and may be able to construct more accurate biological age models (91, 98). New technologies and methods are essential in identifying more novel and accurate biomarkers.

Some of the issues also faced in screening aging markers include the selection of the population; the impact of disease on aging markers; whether there can be a better reference benchmark than CA; the influence of unexpected random events such as unpredictability on the aging rate and predicted life expectancy and mortality; and how to better screen out aging markers by advances in the development of statistical methods and the wide application of artificial intelligence and machine learning. With the advancement of cognitive, technical and statistical models, the screening criteria for aging markers will further progress and improve.

In this review, 11 criteria for screening aging markers are summarized comprehensively and systematically, and the criteria are divided into three aspects according to the content; some possible problems of the criteria are discussed; and the frequency of using the criteria is compared; an evaluation framework is developed and categorized into three levels based on their importance, which is helpful to assess the extent to which a candidate biomarker may be feasible, valid, and useful for a specific context of use. However, some limitations of this review should be mentioned. The framework and three levels proposed in this paper are more based on our previous experiences in constructing population biological age studies and our subjective views on these criteria, and their scientificity and practicability need to be further tested. Currently, there has yet to be a consensus on the criteria for screening aging markers. Establishing an aging marker screening system helps screen aging markers in the aging population.

## Author contributions

YH: Writing – original draft, Conceptualization, Investigation, Methodology. ZL: Conceptualization, Investigation, Methodology, Writing – original draft. YN: Investigation, Resources, Writing – review & editing. YD: Conceptualization, Investigation, Methodology, Writing – original draft. QW: Investigation, Writing – review & editing. XL: Investigation, Writing – review & editing. ZD: Investigation, Writing – review & editing. YZ: Funding acquisition, Investigation, Writing – review & editing. YC: Funding acquisition, Resources, Writing – review & editing. YW: Resources, Writing – review & editing. DZ: Investigation, Writing – review & editing. XS: Resources, Supervision, Writing – review & editing. GC: Resources, Supervision, Writing – review & editing. ZF: Conceptualization, Funding acquisition, Supervision, Writing – review & editing. WZ: Conceptualization, Funding acquisition, Supervision, Writing – review & editing. XC: Conceptualization, Funding acquisition, Supervision, Writing – review & editing.
