# Supplementary material for: Progress in the study of aging marker criteria in human populations
Source: Front Public Health. 2024 Jan 24;12:1305303. doi: 10.3389/fpubh.2024.1305303 (PMC10847233; doi:10.3389/fpubh.2024.1305303)
Supplement: Supplementary file 1 [file Table_1.docx]

**Supplementary Table 1**

Summary of criteria for aging markers in different literatures.

The contents in the table are the criteria for screening aging markers in the previous literatures.

| **Number** | **Authors** | **Year** | **Criteria** |
| --- | --- | --- | --- |
| 1 | Strehler B.(1) | 1962 | - Changes advancing age should be cumulative, progressive, intrinsic, deleterious to biologic function, and universal. |
| 2 | Hollingsworth, J. W.(2) | 1965 | - Showed significant change with age, measured an independent function and were not too highly correlated with another biomarker; - They were reliable "physiologic age" were it possible to demonstrate that this measure was more closely related to subsequent, age dependent changes than chronologic age itself; - Related to mortality. |
| 3 | Damon, A.(3) | 1972 | - Showed significant change with age, that they measured an independent function and were not too highly correlated with another biomarker. |
| 4 | Ries, W.(4) | 1974 | - Covered essential areas of human functional capacity; they were quantitative. |
| 5 | Furukawa, T.(5) | 1975 | - Reflected physical fitness. |
| 6 | Webster, Ian W.(6) | 1976 | - Showed significant change with age; - They measured an independent function and were not too highly correlated with another biomarker. |
| 7 | Borkan, G.A.(7) | 1980 | - Showed significant change with age; - They covered a range of physical functions, that they had been used in previous studies; not restricted to deleterious aspects of aging. |
| 8 | Nakamura, E.(8) | 1985 | - Covered a range of physical functions; easy to calculate and measure. |
| 9 | Ringel, R. L.(9) | 1987 | - Covered a range of physical functions; readily observable |
| 10 | Baker, G.T.(10) | 1988 | - Reflect basic biological process of aging rather than diseases; - Have high reproducibility in cross-species comparisons; - Change independently of passage of time; nonlethal, Noninvasive or minimally invasive; - Be measurable during a relatively short time interval. |
| 11 | McClearn, G. E. (11) | 1990 | - Possible existence of one or a few major “pacemakers” of aging; - Subprocesses are influenced by a sufficiently large number of genetic loci that they cannot be effectively studied by Mendelian methods; - Warrant a lifespan examination of predictability of late life changes from earlier states. |
| 12 | Mooradian, A. D.(12) | 1990 | - Quantitative correlation with biologic parameter and age of subjects; - Not altered by disease process; - Factors that modulate the aging rate should appropriately alter the putative biomarker, Generalizability of the biomarker to different syndromes of premature aging; - Noninvasive or minimally invasive; - Reliable changes within relatively short interval of time compared to life span. |
| 13 | Robert, A.(13) | 1991 | - Change with time reflects the rate of aging; - Reflect physiological age; - Be monitoring some basic, important process; - Be crucial to the maintenance of health; - Serve as predictor of lifespan and/or serve as a retrospective marker of aging; - Be highly reproducible; - Display change over a relatively short period; - Be measurable in a variety of species; - Noninvasive or minimally invasive. |
| 14 | Nakamura, E.(14) | 1994 | - Significant cross-sectional correlation with age; - Significant longitudinal change with age consistent with the cross sectional; - Significant stability of individual differences; - Rate of age-related change proportional to differences in lifespan among related species. |
| 15 | McClearn, G. E.(15) | 1997 | - Should change at a rate reflecting the rate of aging; - Reflect physiological age; - Participate in a basic causal process; - Health related; - Predict age at death; - Highly reproducible; - Display change over a relatively short period; - Measurable in a variety of species; - Noninvasive or minimally invasive. |
| 16 | Nakamura, E.(16) | 1998 | - Cross-sectional correlation with CA; - Longitudinal change same direction with the cross-sectional correlation; - Reliability as indicated by stability of individual differences over time; - Significant loading onto a principal component accounting for a high degree of covariance both when CA was and was not included as a variable. |
| 17 | Miller, R.A.(17) | 2001 | - Predict the outcome of a wide range of age-sensitive tests in multiple Physiological domains, in an age-coherent way, and do so better than chronological age; - Should predict remaining longevity at an age at which 90% of the population is still alive; - Should not alter either life expectancy or the outcome of subsequent tests of other age-sensitive traits. |
| 18 | Ueno, L. M.(18) | 2003 | - Significant cross-sectional correlation with age; - Significant longitudinal change with age consistent with the cross-sectional correlation; - No obvious redundancy with other selected variables; - Rate of age-related change proportional to differences in lifespan among related species. |
| 19 | Butler, R. N.(19) | 2004 | - Should predict the outcome of a wide range of age-sensitive tests in multiple physiological and behavioral domains, in an age-coherent way, and do so better than chronological age; - Predict remaining longevity at an age at which 90% of the population is still alive, and do so for most of the specific illnesses that afflict the species under study; - Should not alter life expectancy or the outcome of subsequent tests of other age-sensitive tests. |
| 20 | Karasik, D.(20) | 2005 | - Predict, better than chronological age, declines in one or several organ systems and/or the likelihood of a disease event; - Be highly correlated with age-related loss of function, rather than only being correlated with chronological age or survival; - Noninvasive or minimally invasive, reliably measured, preferably on a quantitative scale. |
| 21 | Parentini, I.(21) | 2005 | - Quantitative correlation with age in all tissues and are not altered by several age-dependent diseases in the same direction as that of aging; - Altered appropriately by factors that modulate the aging rate like caloric restriction and physical exercise; - Applicable to different tissues across mammalian species. |
| 22 | Johnson, T. E.(22) | 2006 | - Predict the rate of aging; - Be a better predictor of life span than chronological age. - It must monitor a basic process that underlies the aging process, not the effects of disease; - Tested repeatedly; - Noninvasive or minimally invasive; - Works in humans and in laboratory animals; - This is so that it can be tested in lab animals before being validated in humans. |
| 23 | Crews, D. E.(23) | 2007 | - Should be relatively poorly correlated with chronological age not time dependent; - Should not be correlated with chronological age nor should it be based on age; - As aspects of cellular and physiological function whose alteration, appearance, or disappearance coincided with specific aspects of senescent biology; - Scale with senescent change; Lead the development of interventions for senescent processes. |
| 24 | Nakamura, E.(24) | 2007 | - Significant cross-sectional correlation with age; - Significant longitudinal change with age consistent with the cross-sectional correlation; - Significant stability of individual differences; - Rate of age-related change proportional to differences in life span among related species. |
| 25 | Malkin, I, L.(25) | 2007 | - It reflects the intensity of an individual’s skeletal changes in relation to its coevals of the same sex, and the range of the index does not depend on the age cohort. |
| 26 | Morrow, D. A. (26) | 2007 | - Clinician can measure it; add new information; and it help the clinician manage patients. |
| 27 | Swindell, W. R.(27) | 2008 | - Be able to distinguish among individuals who are aging at different rates; - Predict the outcome of a wide range of age-sensitive events in different physiological systems and, ultimately, should be predictive of individual life span. |
| 28 | Hlatky, M. A.(28) | 2009 | - Association between the marker and as a predictor of future events; - New markers should provide incremental prognostic information; - Simpler or safer to measure, be more reproducible; measurement is simple, inexpensive, and readily available，provide incremental prognostic information to be of clinical value the new factor should be tested for significance; - More outcome events are needed to provide adequate statistical power for the test. |
| 29 | Park, J.(29) | 2009 | - Should reflect specific human functions which change uniformly not due to disease processes, but due to the simple aging process in the normally aging person; - Study subjects should not have diseases or functional abnormalities which exceeded what could be expected from a simple aging process; - Functional decrements or diseases as a part of normal aging process. |
| 30 | Sprott, R. L.(30) | 2010 | - Needs disease free subjects; - Biomarkers of aging is the validity of the concept of basic biological aging processes and that there are therefore, biological parameters that will be better measures of rate of aging than chronological age. |
| 31 | Vasto, S.(31) | 2010 | - Reflect some basic property of aging; - Be reproducible in cross-species comparison; - Change independently of the passage of chronological time; Be obtainable by noninvasive means; Be measurable during a short interval of life span. |
| 32 | Simm, A.(32) | 2010 | - Predict the rate of ageing; Be a better predictor of life span than chronological age alone; - Monitor a basic process that underlies the ageing process, not the effects of disease; - Be able to be tested repeatedly without harming the person; - Be something that works in humans and in laboratory animals, can be tested in lab animals before being validated in humans. |
| 33 | Majkić-Singh, N.(33) | 2011 | - Investigated in the general and the observed population, and if there are variations depending on the basic demographic characteristics such as gender, age and race, which may impose certain limitations on their applicability; - The investigated biomarker correlates with the known risk factors or reflects other pathophysiological mechanisms that participate in the investigated disease; - Its inclusion increased the prognostic value of the existing prognostic models. |
| 34 | AFfAR A (34) | 2016 | - Predict a person’s function in an age-related way; - Indepen­dently of chronological age to predict the onset of age-related diseases; Testable and not harmful; - Technically simple most clinical labora­tories could perform the test; Should work in laboratory animals as well as humans. |
| 35 | Ding, J.(35) | 2011 | - Be identified which can be predictive of the aging process; has biological characteristics (e.g., physiological and molecular) that change as a function of age and, therefore, are indicative of a given age period; - One of the ultimate goals of the aging field is to develop therapeutic agents that may extend human lifespan, and, perhaps more importantly, improve the quality of life or ‘health span; declines during normal aging in humans; - Lastly, humans and other animals including mice show declined physical activity as they age. |
| 36 | Kimura, M.(36) | 2012 | - Significant cross-sectional correlation with age; - Significant longitudinal change with age consistent with the cross-sectional correlation; - Significant stability of individual differences; - The rate of the age-related change was proportional to differences in lifespan among related species. |
| 37 | Jee, H.(37) | 2012 | - Predict for the rate at which a person is aging; - Reflect the basic biological process of aging; - Monitor a process central to biology of aging, instead of the biology of a specific disease; have high reproducibility in cross-species comparisons of functional age and change independently with the passage of time and reflect functional age; - Observe the intrinsic biological progression of aging, abnormal physiological or functional factors should be excluded; - Be able to best tested repeatedly and not cause harm to an individual; Acts in both humans and laboratory animals. |
| 38 | Mishra, Showalter.(38) | 2012 | - A biomarker should predict for the rate at which a person is aging; - The biomarker should monitor a process central to biology of aging, instead of the biology of a specific disease; - The biomarker should be able to best tested repeatedly and not cause harm to an individual; - A biomarker must be something that acts in both humans and laboratory animals; - Implementated in in disease-free individuals. |
| 39 | Engelfriet, P. M.(39) | 2013 | - Predict the rate of aging, monitor a basic mechanism of aging process, not an effect of disease. |
| 40 | López-Otín, C.(40) | 2013 | - Should manifest during normal aging; - Experimental aggravation should accelerate aging; - Experimental amelioration should retard the normal aging process and hence increase healthy lifespan. |
| 41 | Le Couteur,  D. G.(41) | 2013 | - Predict the rate of aging; - A better predictor of life span; - Monitor a basic process of the aging process, not effect by disease; - Tested repeatedly without harming the person or animal; - Works in human and in laboratory animal; - Easy to measure and change over a relatively short period of time. |
| 42 | Zhang, W-G.(42) | 2014 | - High repeatability; - Significant differences among individuals, reflect changes of the aging rate in a relatively short time; - Show no change due to disease; - Noninvasive or minimally invasive; - Can repeated testing. |
| 43 | [MartinRuiz, C.](#_ENREF_30) (43) | 2014 | - Should be inexpensive, fast, and show minimal methodological variation within and between laboratories. |
| 44 | Cohen, A. A.(44) | 2015 | - Common; - Used often in clinic; - Cheap, increasing the relevance of any results for clinical implementation. |
| 45 | Deelen, J.(45) | 2016 | - Shows a change with chronological age; - Discriminates individuals based on their familial propensity for longevity, and associates with known health parameters and morbidity and/or mortality in prospective studies； - Biomarker (of healthy ageing) should be inexpensive, fast, and show minimal methodological variation within and between laboratories. |
| 46 | Arbeev, K. G.(46) | 2016 | - Repeatable; - Easily obtainable; - Expected to be better predictors of mortality and health-related outcomes than individual biomarkers. |
| 47 | Ylhävä, J.(47) | 2017 | - It must predict the rate of aging. In other words, it would tell exactly where a person is in their total life span. It must be a better predictor of life span than chronological age; - It must monitor a basic process that underlies the aging process, not the effects of disease; - It must be able to be tested repeatedly without harming the person. For example, a blood test or an imaging technique. - It must be something that works in humans and in laboratory animals, such as mice. This is so that it can be tested in lab animals before being validated in humans. Independent of chronological age in humans. |
| 48 | Justice, J. N.(48) | 2018 | - Measurement reliability and feasibility; - Represent biologic aging processes. The biomarker should have face validity such that it represents a process or processes relevant to biologic aging hallmarks, and changes in a measurable and consistent manner with chronological age; - Robust and consistent association with risk of death, and clinical/functional trial endpoints. Association with risk; - Responsive to intervention. A biomarker of aging for geroscience-guided trials should be responsive to interventions that affect the biology of aging ideally over a relatively short period of time. A quick response to intervention would allow for shorter trials for fully vetted biomarkers. |
| 49 | Levine, M. E.(49) | 2018 | - Produce realistic measurements, within the limits of recorded life span; - Be able to identify at-risk individuals prior to entering a disease state; - Be a better predictor of multiple age-associated biological and functional outcomes than is chronological age; - Predict both remaining longevity and disease-specific mortality in a population of which 90 % of the individuals are still alive; - Be a better predictor of all-cause and disease-specific mortality when compared with chronological age. |
| 50 | Cole, J. H.(50) | 2019 | - Predict the rate of ageing, (i.e., inform where a person is in their total lifespan). It must be a better predictor of lifespan than chronological age; - Monitor a basic process that underlies the ageing process, not the effects of disease; - Can be tested repeatedly without causing harm; - Work in humans and in laboratory animals, so it can be tested in animals prior to validation in humans. |
| 51 | Dodig, S.(51) | 2019 | - They have to predict the rate of aging; - Monitor a basic process that underlies the aging process, be able to be tested repeatedly without harming the person; - In addition, biomarkers have to be indicators of biological processes, pathogenic processes or pharmacological responses to therapeutic intervention; |
| 52 | Rahman, S. A.(52) | 2019 | - Biological age provides a better measure of the life expectancy of an individual than his or her chronological age; - Longitudinal repeated measures are needed that track change over time; - They also used chronological age as an input parameter to predict BA; |
| 53 | Gialluisi, A.(53) | 2019 | - Predicts All-Cause Mortality and shows the best performances in terms of accuracy and prediction of mortality risk. |
| 54 | Earls, J. C.(54) | 2019 | - Has been proposed to better predict mortality and disease than chronological age (CA). |
| 55 | Waziry, R.(55) | 2019 | - Biomarkers were selected based on their independent correlation with chronological age. All the individual biomarkers were age-dependent; - Biological age showed a stronger association with the following end-points (diabetes mellitus, stroke, cancer and mortality. Biological age, calculated at baseline was able to predict, better than chronological age alone and traditional biomarkers, mortality, morbidity and onset of specific diseases such as stroke and cancer; - Can be reproduced. |
| 56 | Schultz, M. B.(56) | 2020 | - Should be non-invasive to allow for repeated measurements without altering the health or lifespan of the animal measured; - Any useful biometric or biomarker for biological age should track with chronological age and should serve as a better predictor of remaining longevity health and other age-associated outcomes than does chronological age alone, even at an age when most of a population is still alive; - Cost-effective; - Which accurately predicts life expectancy and the efficacy of a lifespan-extending intervention up to a year in advance. |
| 57 | Solovev, I.(57) | 2020 | - Must change with age; Have to predict mortality better than chronological age; Allow foreseeing the early stages of a specific age-related disease; To be minimally invasive do not require serious intervention or painful procedure; - To be sensitive to early signs of aging (as opposed to frailty and mortality, which are too late for prevention and geroprotection); Have predictability with collecting in the foreseeable time range; Have low analytical variability (robustness and reproducibility). |
| 58 | Belsky, D. W.(58) | 2020 | - A useful measure should be non-invasive, inexpensive, reliable, and highly sensitive to biological change; - The potential of simplifying aging rate measurements into an exportable, inexpensive and non-invasive blood test is illustrated, thereby simplifying the implementation of aging rate measurements in studies of interventions to slow down the biological aging process. |
| 59 | Nelson, P. G.(59) | 2020 | - Biomarkers can inform diagnosis and associated prognosis. Biological quantities that are convenient to measure, and that give us medically important information; - Biomarkers can reveal disease mechanisms; - A biomarker can provide information that enhances; - Interventions and improves outcomes. To be non-causative. |
| 60 | Miller, B.(60) | 2020 | - Biomarkers that reflect aging could be used to target age-related diseases with precision and monitor treatment efficacy. |
| 61 | Rivero-Segura, N. A(61) | 2020 | - Aging biomarkers reflect the physiological state of individuals and the underlying mechanisms related to homeostatic changes throughout an individual lifespan. |
| 62 | Zhavoronkov, A.(62) | 2020 | - Modifiable; - Features that are predictive but not modifiable, such as the age of death of parents and/or grandparents, should be excluded. This increases the number of actionable items available to the model users; - Non-leaking. Age of children, years in retirement, and other demographic questions directly related to the chronological age were excluded. Such variables; - Are trivial in interpretation and rarely lead to therapy targets. Moreover, they may obscure valuable, therapeutic trends by having disproportionately large importance scores; - Predictive. A feature needs to be associated with age-related changes. Selecting predictive features can be realized as an iterative process, in which feature; - Importance analysis is carried out after each round of model training. In the case of MIDUS 1 most predictive variables were associated with health, personality traits, occupation and other psychosocial aspects of life; - Robust. Aging clock features need to be predictive across different demographics, as well as in the same person at different time points; - Non-collinear. Features that are strongly correlated with each other should be reduced. For example, such variables as weight, body mass index, and waist circumference are strongly correlated and add little predictive value when present in an aging clock all at once. |
| 63 | Guerville, F.(63) | 2020 | The American Federation of Aging Research (AFAR) defined 3 criteria that a biomarker of aging should ideally meet:   - Mark the individual stage of aging and predict mortality better than chronological age; - Monitor aging in a range of systems and not the effects of diseases; - Allow longitudinal non-invasive tracking in animals and humans.   For this narrative review, our search for putative biomarkers of healthy aging was based on the following criteria:   - Associated with survival, aging-related diseases, frailty and/or functional loss; - Putative biomarkers should have been studied in humans. Whenever available, animal data were also considered; - It should manifest during normal aging, its experimental aggravation should accelerate aging and its experimental amelioration should delay the normal aging process and thus increase healthy aging. Thus, there is causal evidence for the implication of these biological mechanisms in the aging process, and associated therapeutic potential; - Chose non-invasive biomarkers; - Measuring aging in a range of systems, non-invasively in humans and animals, predicting mortality, age-related diseases and loss of functions. |
| 64 | Colloca, G.(64) | 2020 | The American Federation for Aging Research (AFAR) recommends the following criteria for biomarkers of aging：   - It must predict a person’s physiological, cognitive, and physical function in an age-related way, independently of chronological age; - It must be testable and not harmful to test subjects (for example a blood test or an imaging technique); it must also be technically simple to perform, and it must be accurate and reproducibly without the need for specialized equipment or techniques; - It should work in laboratory animals as well as humans since preliminary testing is always done in nonhuman subjects. |
| 65 | Kwon, D.(65) | 2021 | - Biological aging measures are correlated with chronological age; - Biological aging measures are correlated with one another. - Biological aging measures are tested for association with health span related characteristics: mortality, disability, physical function, and self-rated health; - A set of analyses tests socioeconomic patterning of biological aging algorithms; - Can predict the healthy lifespan. |
| 66 | Armanious, K.(66) | 2021 | - For the prediction of the mortality risk. Other methods incorporate the ca as ground truth labels and examine the relation between the predicted ages and other health indicators. |
| 67 | Verschoor, C. P.(67) | 2021 | - Highly standardized, minimally invasive, and/or quick to obtain; - Significantly associated with the hazard of all-cause mortality, as well as cardiovascular and cancer-specific mortality. |
| 68 | Hastings, W. J.(68) | 2021 | - Correlated with chronological age and displayed superior mortality and morbidity prediction; - Measures of this sort are predictive of morbidity and mortality, show variation by young adulthood, and appear responsive to intervention; - Can predict physical and cognitive function and predict healthy life expectancy. |
| 69 | Ashiqur Rahman, S.(69) | 2021 | - Provide a better measure of the life expectancy of an individual than his or her CA; - Based on some age-dependent variables; - Showed that different anthropometric attributes are correlated with age and thus used them to predict all-cause mortality. |
| 70 | Gialluisi, A.(70) | 2021 | - Showed higher accuracy in predicting chronological age and mortality risk. |
| 71 | Verschoor, C. P.(71) | 2021 | - Reliably predict numerous age-related outcomes such as cardiovascular disease risk, depression, post-operative recovery and death. |
| 72 | Zhong, X. (72) | 2021 | - Accurate measure of the rate of human aging; - The BA estimates robustly predicted frailty and mortality and outperformed ca; - Ba is better than ca for measuring life span (mortality) and health span (frailty); - A greater BA was associated with more adverse socio-behavioral risk factors and health status, and vice versa; - Predict future risks of adverse functional and mortality outcome. |
| 73 | Kuo, C. L.(73) | 2021 | - Biological age measures outperform chronological age in predicting various aging outcome; - A valid BA measure needs to outperform chronological age in predicting lifespan and a wide range of age-sensitive tests in multiple physiological and behavioral domains. |
| 74 | Ladejobi,  Adetola O.(74) | 2021 | - Biological age, also referred to as physiologic age, on the other hand, refers to the gradual decline in an organism’s functional status—the clearest measure of which is mortality; - Discrepancies between AI-enabled ECG predicted and actual chronological age were associated with individual health status; - Associated with all-cause and cardiovascular mortality, beyond what would be expected from chronological age alone. |
| 75 | Bae, Chul-Young.(75) | 2021 | - Based on biomarkers identified for high correlation with age; - BA measured in relatively healthy adults better reflects their actual health status than CA does; - A simple linear regression analysis was performed to find out the linear relationship between the predicted BA and CA. |
| 76 | Lohman, T.(76) | 2021 | - Predict remaining lifespan, mortality risk, and age-related morbidity risk; - The biomarker should predict the outcome of a wide range of age-sensitive tests in multiple physiological and behavioral domains, in an age-coherent way, and do so better than chronological age; - It should predict remaining longevity at an age at which 90% of the population is still alive and do so for most of the specific illnesses that afflict the species under study; - Its measurement should not alter life expectancy or the outcome of subsequent tests of other age-sensitive tests.   The American Federation for Aging Research (AFAR) formulated the criteria for aging biomarkers as follows:   - It must predict the rate of aging. In other words, it would tell exactly where a person is in their total life span. It must be a better predictor of life span than chronological age; - It must monitor a basic process that underlies the aging process, not the effects of disease; - It must be able to be tested repeatedly without harming the person. For example, a blood test or an imaging technique; - It must be something that works in humans and in laboratory animals, such as mice. This is so it can be tested in lab animals before being validated in humans. |
| 77 | Green, S.(77) | 2021 | - A measurable indicator of health conditions (Individuals without disease) that can inform disease diagnosis, treatment, or prevention. |
| 78 | Nie, C.(78) | 2022 | - Assess the true aging rate; - Constructing BA clocks is predicting abnormalities, disorders, lifespan and even mortality. |
| 79 | Belsky, D. W.(79) | 2022 | - Associate with morbidity, disability, and mortality and assess the aging rate; - High test- retest reliability. |
| 80 | Drewelies, J.(80) | 2022 | - Biological age must be able to predict the rate of aging and it must be a better predictor of life span than chronological age; - In addition, biological age must have the capability to monitor one or more basic processes that contribute to or underlie aging rather than merely representing effects of disease, be tested without harming the person, and work in humans and laboratory animals alike; - Suggest that a marker of biological age must predict existing and prospective age-associated phenotypes over and above chronological age; - Reflect the rate of aging and associate with mortality hazards, morbidity, and physical functioning; - Convenient and non-invasive, inexpensive, standard validated. |
| 81 | Nusinovici, S.(81) | 2022 | - Deep learning can predict biological age, providing a novel alternative method for measuring aging and predicting mortality, cardiovascular disease and cancer risk. |
| 82 | Li, Z.(82) | 2023 | - Significantly correlated with CA, non-redundant variables; - Monitors the underlying mechanisms of the aging process rather than the effects of disease, reflects different organs or physiological functions, repeatable measurements; - Variables with higher loadings within the first principal component, test results can be quantified. |
| 83 | López-Otín, C.(83) | 2023 | - Their age-associated manifestation; - The acceleration of aging by experimentally accentuating them; - The opportunity to decelerate, stop, or reverse aging by therapeutic interventions on them. |
| 84 | Li, Z.(84) | 2023 | - Consider that aging biomarkers are linearly correlated with CA; - Biomarkers of aging, which, in addition to having good reliability, are generally screened in large populations and used by other investigators. |
| 85 | Moqri, M.(85) | 2023 | - Measurement of the biomarker should be minimally invasive and reliable, i.e., it should be possible to conduct longitudinal measurements with little technical variability; - The biomarker should be relevant to aging; - The biomarker should predict functional aspects of aging, e.g., mortality, better than chronological age; - The biomarker should be responsive to longevity interventions. |

**References**

1. Strehler B. *Time, Cells, and Aging.* Bernard L. Strehler Academic Press (1962).

2. Hollingsworth JW, Hashizume A, Jablon S. Correlations between Tests of Aging in Hiroshima Subjects--an Attempt to Define" Physiologic Age". The Yale journal of biology and medicine. (1965) 38:11.

3. Damon A. Predicting Age from Body Measurements and Observations. The International Journal of Aging and Human Development. (1972) 3:169-73. doi: 10.2190/WYAP-U442-NAD1-8FVP.

4. Ries W. Problems Associated with Biological Age. Experimental gerontology. (1974) 9:145-9. doi: 10.1016/0531-5565(74)90044-8.

5. Furukawa T, Inoue M, Kajiya F, Inada H, Takasugi S, Fukui S, et al. Assessment of Biological Age by Multiple Regression Analysis. Journal of gerontology. (1975) 30:422-34. doi: 10.1093/geronj/30.4.422

6. Webster IW, Logie AR. A Relationship between Functional Age and Health Status in Female Subjects. Journal of gerontology. (1976) 31:546-50. doi: 10.1093/geronj/31.5.546.

7. Borkan GA, Norris AH. Assessment of Biological Age Using a Profile of Physical Parameters. Journal of gerontology. (1980) 35:177-84. doi: 10.1093/geronj/35.2.177

8. Nakamura E. The Assessment of Physological Age Based Upon a Principal Component Analysis of Various Physiological Variables, J. Kyoto Pref Univ Med. (1985) 94:757-69.

9. Ringel RL, Chodzko-Zajko WJ. Vocal Indices of Biological Age. Journal of Voice. (1987) 1:31-7. doi: 10.1016/S0892-1997(87)80021-8.

10. Baker GT, 3rd, Sprott RL. Biomarkers of Aging. Exp Gerontol. (1988) 23:223-39. doi: 10.1016/0531-5565(88)90025-3.

11. McClearn GE. Biomarker characteristics and research on the genetics aging. New Jersey: The Telford Press (1990). 233-254p.

12. Mooradian AD. Biomarkers of Aging: Do We Know What to Look For? Journal of Gerontology. (1990) 45:B183-6. doi: 10.1093/geronj/45.6.b183.

13. Robert A. *Biology of Aging: Observations and Principles*. Engle-wood Cliffs: New Jersey: Prentice—Hall Inc (1991).doi: 10.1016/1043-2760(92)90114-G.

14. Nakamura E, Lane MA, Roth GS, Cutler RG, Ingram DK. Evaluating Measures of Hematology and Blood Chemistry in Male Rhesus Monkeys as Biomarkers of Aging. Experimental gerontology. (1994) 29:151-77. doi: 10.1016/0531-5565(94)90048-5.

15. McClearn GE. Biomarkers of Age and Aging. Experimental gerontology. (1997) 32:87-94. doi: 10.1016/s0531-5565(96)00067-8.

16. Nakamura E, Lane MA, Roth GS, Ingram DK. A Strategy for Identifying Biomarkers of Aging: Further Evaluation of Hematology and Blood Chemistry Data from a Calorie Restriction Study in Rhesus Monkeys. Experimental gerontology. (1998) 33:421-43. doi: 10.1016/s0531-5565(97)00134-4.

17. Miller RA. Biomarkers of Aging. Sci Aging Knowledge Environ. (2001) 2001:pe2. doi: 10.1126/sageke.2001.1.pe2.

18. Ueno LM, Yamashita Y, Moritani T, Nakamura E. Biomarkers of Aging in Women and the Rate of Longitudinal Changes. Journal of physiological anthropology and applied human science. (2003) 22:37-46. doi: 10.2114/jpa.22.37.

19. Butler RN, Sprott R, Warner H, Bland J, Feuers R, Forster M, et al. Biomarkers of Aging: From Primitive Organisms to Humans. J Gerontol A Biol Sci Med Sci. (2004) 59:B560-7. doi: 10.1093/gerona/59.6.b560.

20. Karasik D, Demissie S, Cupples LA, Kiel DP. Disentangling the Genetic Determinants of Human Aging: Biological Age as an Alternative to the Use of Survival Measures. The Journals of Gerontology Series A: Biological Sciences and Medical Sciences. (2005) 60:574-87. doi: 10.1093/gerona/60.5.574.

21. Parentini I, Cavallini G, Donati A, Gori Z, Bergamini E. Accumulation of Dolichol in Older Tissues Satisfies the Proposed Criteria to Be Qualified a Biomarker of Aging. The Journals of Gerontology Series A: Biological Sciences and Medical Sciences. (2005) 60:39-43. doi: 10.1093/gerona/60.1.39.

22. Johnson TE. Recent Results: Biomarkers of Aging. Experimental gerontology. (2006) 41:1243-6. doi: 10.1016/j.exger.2006.09.006.

23. Crews DE. Senescence, Aging, and Disease. Journal of physiological anthropology. (2007) 26:365-72. doi: 10.2114/jpa2.26.365.

24. Nakamura E, Miyao K. A Method for Identifying Biomarkers of Aging and Constructing an Index of Biological Age in Humans. The Journals of Gerontology Series A: Biological Sciences and Medical Sciences. (2007) 62:1096-105. doi: 10.1093/gerona/62.10.1096.

25. Malkin I, Kalichman L, Kobyliansky E. Heritability of a Skeletal Biomarker of Biological Aging. Biogerontology. (2007) 8:627-37. doi: 10.1007/s10522-007-9104-8

26. Morrow DA, de Lemos JA. Benchmarks for the Assessment of Novel Cardiovascular Biomarkers. Circulation. (2007) 115:949-52. doi: 10.1161/CIRCULATIONAHA.106.683110.

27. Swindell WR, Harper JM, Miller RA. How Long Will My Mouse Live? Machine Learning Approaches for Prediction of Mouse Life Span. The Journals of Gerontology Series A: Biological Sciences and Medical Sciences. (2008) 63:895-906. doi: 10.1093/gerona/63.9.895.

28. Hlatky MA, Greenland P, Arnett DK, Ballantyne CM, Criqui MH, Elkind MS, et al. Criteria for Evaluation of Novel Markers of Cardiovascular Risk :A Scientific Statement from the American Heart Association. Circulation. (2009) 119:2408-16. doi: 10.1161/CIRCULATIONAHA.109.192278.

29. Park J, Cho B, Kwon H, Lee C. Developing a Biological Age Assessment Equation Using Principal Component Analysis and Clinical Biomarkers of Aging in Korean Men. Archives of Gerontology and Geriatrics. (2009) 49:7-12. doi: 10.1016/j.archger.2008.04.003

30. Sprott RL. Biomarkers of Aging and Disease: Introduction and Definitions. Experimental gerontology. (2010) 45:2-4. doi: 10.1016/j.exger.2009.07.008.

31. Vasto S, Scapagnini G, Bulati M, Candore G, Castiglia L, Colonna-Romano G, et al. Biomarkes of Aging. Frontiers in bioscience (Scholar edition). (2010) 2:392. doi: 10.2741/s72.

32. Simm A, Johnson TE. Biomarkers of Ageing: A Challenge for the Future. Experimental gerontology. (2010) 45:731-2. doi: 10.1016/j.exger.2010.08.006.

33. Majkić-Singh N. What Is a Biomarker? From Its Discovery to Clinical Application. Journal of Medical Biochemistry. (2011) 30:186-92. doi: 10.2478/v10011-011-0029-z.

34. AFfAR A. Biomarkers of Aging: An Introduction to Aging Science Brought to You by the American Federation for Aging Research. American federation for aging research, New York, NY. (2016).

35. Ding J, Kopchick JJ. Plasma Biomarkers of Mouse Aging. AGE. (2011) 33:291-307. doi: 10.1007/s11357-010-9179-z.

36. Kimura M, Mizuta C, Yamada Y, Okayama Y, Nakamura E. Constructing an Index of Physical Fitness Age for Japanese Elderly Based on 7-Year Longitudinal Data: Sex Differences in Estimated Physical Fitness Age. Age. (2012) 34:203-14. doi: 10.1007/s11357-011-9225-5.

37. Jee H, Jeon BH, Kim YH, Kim H-K, Choe J, Park J, et al. Development and Application of Biological Age Prediction Models with Physical Fitness and Physiological Components in Korean Adults. Gerontology. (2012) 58:344-53. doi: 10.1159/000335738.

38. Mishra MV, Showalter TN, Dicker AP. Biomarkers of Aging and Radiation Therapy Tailored to the Elderly: Future of the Field. Seminars in Radiation Oncology. (2012) 22:334-8. doi: 10.1016/j.semradonc.2012.05.010.

39. Engelfriet PM, Jansen EH, Picavet HSJ, Dollé ME. Biochemical Markers of Aging for Longitudinal Studies in Humans. Epidemiologic reviews. (2013) 35:132-51. doi: 10.1093/epirev/mxs011.

40. López-Otín C, Blasco MA, Partridge L, Serrano M, Kroemer G. The Hallmarks of Aging. Cell. (2013) 153:1194-217. doi: 10.1016/j.cell.2013.05.039.

41. Le Couteur DG, Simpson SJ, de Cabo R. Are Glycans the Holy Grail for Biomarkers of Aging? The Journals of Gerontology Series A: Biological Sciences and Medical Sciences. (2014) 69:777-8. doi: 10.1093/gerona/glt202.

42. Zhang W-G, Bai X-J, Sun X-F, Cai G-Y, Bai X-Y, Zhu S-Y, et al. Construction of an Integral Formula of Biological Age for a Healthy Chinese Population Using Principle Component Analysis. The journal of nutrition, health & aging. (2014):1-6. doi: 10.1007/s12603-013-0345-8.

43. Martin-Ruiz C, von Zglinicki T. Biomarkers of Healthy Ageing: Expectations and Validation. Proc Nutr Soc. (2014) 73:422-9. doi: 10.1017/s0029665114000147.

44. Cohen AA, Milot E, Li Q, Bergeron P, Poirier R, Dusseault-Bélanger F, et al. Detection of a Novel, Integrative Aging Process Suggests Complex Physiological Integration. PLoS One. (2015) 10:e0116489. doi: 10.1371/journal.pone.0116489.

45. Deelen J, van den Akker EB, Trompet S, van Heemst D, Mooijaart SP, Slagboom PE, et al. Employing Biomarkers of Healthy Ageing for Leveraging Genetic Studies into Human Longevity. Exp Gerontol. (2016) 82:166-74. doi: 10.1016/j.exger.2016.06.013.

46. Arbeev KG, Ukraintseva SV, Yashin AI. Dynamics of Biomarkers in Relation to Aging and Mortality. Mech Ageing Dev. (2016) 156:42-54. doi: 10.1016/j.mad.2016.04.010.

47. Jylhävä J, Pedersen NL, Hägg S. Biological Age Predictors. EBioMedicine. (2017) 21:29-36. doi: 10.1016/j.ebiom.2017.03.046.

48. Justice JN, Ferrucci L, Newman AB, Aroda VR, Bahnson JL, Divers J, et al. A Framework for Selection of Blood-Based Biomarkers for Geroscience-Guided Clinical Trials: Report from the Tame Biomarkers Workgroup. Geroscience. (2018) 40:419-36. doi: 10.1007/s11357-018-0042-y.

49. Levine ME, Crimmins EM. Is 60 the New 50? Examining Changes in Biological Age over the Past Two Decades. Demography. (2018) 55:387-402. doi: 10.1007/s13524-017-0644-5.

50. Cole JH, Marioni RE, Harris SE, Deary IJ. Brain Age and Other Bodily 'Ages': Implications for Neuropsychiatry. Mol Psychiatry. (2019) 24:266-81. doi: 10.1038/s41380-018-0098-1.

51. Dodig S, Čepelak I, Pavić I. Hallmarks of Senescence and Aging. Biochem Med (Zagreb). (2019) 29:030501. doi: 10.11613/bm.2019.030501.

52. Rahman SA, Adjeroh DA. Deep Learning Using Convolutional Lstm Estimates Biological Age from Physical Activity. Sci Rep. (2019) 9:11425. doi: 10.1038/s41598-019-46850-0.

53. Gialluisi A, Di Castelnuovo A, Donati MB, de Gaetano G, Iacoviello L. Machine Learning Approaches for the Estimation of Biological Aging: The Road Ahead for Population Studies. Front Med (Lausanne). (2019) 6:146. doi: 10.3389/fmed.2019.00146.

54. Earls JC, Rappaport N, Heath L, Wilmanski T, Magis AT, Schork NJ, et al. Multi-Omic Biological Age Estimation and Its Correlation with Wellness and Disease Phenotypes: A Longitudinal Study of 3,558 Individuals. J Gerontol A Biol Sci Med Sci. (2019) 74:S52-s60. doi: 10.1093/gerona/glz220.

55. Waziry R, Gras L, Sedaghat S, Tiemeier H, Weverling GJ, Ghanbari M, et al. Quantification of Biological Age as a Determinant of Age-Related Diseases in the Rotterdam Study: A Structural Equation Modeling Approach. Eur J Epidemiol. (2019) 34:793-9. doi: 10.1007/s10654-019-00497-3.

56. Schultz MB, Kane AE, Mitchell SJ, MacArthur MR, Warner E, Vogel DS, et al. Age and Life Expectancy Clocks Based on Machine Learning Analysis of Mouse Frailty. Nat Commun. (2020) 11:4618. doi: 10.1038/s41467-020-18446-0.

57. Solovev I, Shaposhnikov M, Moskalev A. Multi-Omics Approaches to Human Biological Age Estimation. Mech Ageing Dev. (2020) 185:111192. doi: 10.1016/j.mad.2019.111192.

58. Belsky DW, Caspi A, Arseneault L, Baccarelli A, Corcoran DL, Gao X, et al. Quantification of the Pace of Biological Aging in Humans through a Blood Test, the Dunedinpoam DNA Methylation Algorithm. Elife. (2020) 9. doi: 10.7554/eLife.54870.

59. Nelson PG, Promislow DEL, Masel J. Biomarkers for Aging Identified in Cross-Sectional Studies Tend to Be Non-Causative. J Gerontol A Biol Sci Med Sci. (2020) 75:466-72. doi: 10.1093/gerona/glz174.

60. Miller B, Wan J. Assay Development and Measurement of the Aging Biomarker Humanin. Methods Mol Biol. (2020) 2144:201-9. doi: 10.1007/978-1-0716-0592-9_18.

61. Rivero-Segura NA, Bello-Chavolla OY, Barrera-Vázquez OS, Gutierrez-Robledo LM, Gomez-Verjan JC. Promising Biomarkers of Human Aging: In Search of a Multi-Omics Panel to Understand the Aging Process from a Multidimensional Perspective. Ageing Res Rev. (2020) 64:101164. doi: 10.1016/j.arr.2020.101164.

62. Zhavoronkov A, Kochetov K, Diamandis P, Mitina M. Psychoage and Subjage: Development of Deep Markers of Psychological and Subjective Age Using Artificial Intelligence. Aging (Albany NY). (2020) 12:23548-77. doi: 10.18632/aging.202344.

63. Guerville F, De Souto Barreto P, Ader I, Andrieu S, Casteilla L, Dray C, et al. Revisiting the Hallmarks of Aging to Identify Markers of Biological Age. J Prev Alzheimers Dis. (2020) 7:56-64. doi: 10.14283/jpad.2019.50.

64. Colloca G, Di Capua B, Bellieni A, Fusco D, Ciciarello F, Tagliaferri L, et al. Biological and Functional Biomarkers of Aging: Definition, Characteristics, and How They Can Impact Everyday Cancer Treatment. Curr Oncol Rep. (2020) 22:115. doi: 10.1007/s11912-020-00977-w.

65. Kwon D, Belsky DW. A Toolkit for Quantification of Biological Age from Blood Chemistry and Organ Function Test Data: Bioage. Geroscience. (2021) 43:2795-808. doi: 10.1007/s11357-021-00480-5.

66. Armanious K, Abdulatif S, Shi W, Salian S, Kustner T, Weiskopf D, et al. Age-Net: An Mri-Based Iterative Framework for Brain Biological Age Estimation. IEEE Trans Med Imaging. (2021) 40:1778-91. doi: 10.1109/tmi.2021.3066857.

67. Verschoor CP, Belsky DW, Ma J, Cohen AA, Griffith LE, Raina P. Comparing Biological Age Estimates Using Domain-Specific Measures from the Canadian Longitudinal Study on Aging. J Gerontol A Biol Sci Med Sci. (2021) 76:187-94. doi: 10.1093/gerona/glaa151.

68. Hastings WJ, Almeida DM, Shalev I. Conceptual and Analytical Overlap between Allostatic Load and Systemic Biological Aging Measures: Analyses from the National Survey of Midlife Development in the United States. J Gerontol A Biol Sci Med Sci. (2022) 77:1179-88. doi: 10.1093/gerona/glab187.

69. Ashiqur Rahman S, Giacobbi P, Pyles L, Mullett C, Doretto G, Adjeroh DA. Deep Learning for Biological Age Estimation. Brief Bioinform. (2021) 22:1767-81. doi: 10.1093/bib/bbaa021.

70. Gialluisi A, Santoro A, Tirozzi A, Cerletti C, Donati MB, de Gaetano G, et al. Epidemiological and Genetic Overlap among Biological Aging Clocks: New Challenges in Biogerontology. Ageing Res Rev. (2021) 72:101502. doi: 10.1016/j.arr.2021.101502.

71. Verschoor CP, Lin DTS, Kobor MS, Mian O, Ma J, Pare G, et al. Epigenetic Age Is Associated with Baseline and 3-Year Change in Frailty in the Canadian Longitudinal Study on Aging. Clin Epigenetics. (2021) 13:163. doi: 10.1186/s13148-021-01150-1.

72. Zhong X, Lu Y, Gao Q, Nyunt MSZ, Fulop T, Monterola CP, et al. Estimating Biological Age in the Singapore Longitudinal Aging Study. J Gerontol A Biol Sci Med Sci. (2020) 75:1913-20. doi: 10.1093/gerona/glz146.

73. Kuo CL, Pilling LC, Liu Z, Atkins JL, Levine ME. Genetic Associations for Two Biological Age Measures Point to Distinct Aging Phenotypes. Aging Cell. (2021) 20:e13376. doi: 10.1111/acel.13376.

74. Ladejobi AO, Medina-Inojosa JR, Shelly Cohen M, Attia ZI, Scott CG, LeBrasseur NK, et al. The 12-Lead Electrocardiogram as a Biomarker of Biological Age. European Heart Journal-Digital Health. (2021) 2:379-89. doi: 10.1093/ehjdh/ztab043

75. Bae C-Y, Im Y, Lee J, Park C-S, Kim M, Kwon H, et al. Comparison of Biological Age Prediction Models Using Clinical Biomarkers Commonly Measured in Clinical Practice Settings: Ai Techniques Vs. Traditional Statistical Methods. Frontiers in Analytical Science. (2021):8. doi: 10.3389/frans.2021.709589.

76. Lohman T, Bains G, Berk L, Lohman E. Predictors of Biological Age: The Implications for Wellness and Aging Research. Gerontol Geriatr Med. (2021) 7:23337214211046419. doi: 10.1177/23337214211046419.

77. Green S, Hillersdal L. Aging Biomarkers and the Measurement of Health and Risk. Hist Philos Life Sci. (2021) 43:28. doi: 10.1007/s40656-021-00367-w.

78. Nie C, Li Y, Li R, Yan Y, Zhang D, Li T, et al. Distinct Biological Ages of Organs and Systems Identified from a Multi-Omics Study. Cell Rep. (2022) 38:110459. doi: 10.1016/j.celrep.2022.110459.

79. Belsky DW, Caspi A, Corcoran DL, Sugden K, Poulton R, Arseneault L, et al. Dunedinpace, a DNA Methylation Biomarker of the Pace of Aging. Elife. (2022) 11. doi: 10.7554/eLife.73420.

80. Drewelies J, Hueluer G, Duezel S, Vetter VM, Pawelec G, Steinhagen-Thiessen E, et al. Using Blood Test Parameters to Define Biological Age among Older Adults: Association with Morbidity and Mortality Independent of Chronological Age Validated in Two Separate Birth Cohorts. Geroscience. (2022). doi: 10.1007/s11357-022-00662-9.

81. Nusinovici S, Rim TH, Yu M, Lee G, Tham YC, Cheung N, et al. Retinal Photograph-Based Deep Learning Predicts Biological Age, and Stratifies Morbidity and Mortality Risk. Age Ageing. (2022) 51:afac065. doi: 10.1093/ageing/afac065.

82. Li Z, Zhang W, Duan Y, Niu Y, Chen Y, Liu X, et al. Progress in Biological Age Research. Front Public Health. (2023) 11:1074274. doi: 10.3389/fpubh.2023.1074274.

83. López-Otín C, Blasco MA, Partridge L, Serrano M, Kroemer G. Hallmarks of Aging: An Expanding Universe. Cell. (2023) 186:243-78. doi: 10.1016/j.cell.2022.11.001.

84. Li Z, Zhang W, Duan Y, Niu Y, He Y, Chen Y, et al. Biological Age Models Based on a Healthy Han Chinese Population. Arch Gerontol Geriatr. (2023) 107:104905. doi: 10.1016/j.archger.2022.104905.

85. Moqri M, Herzog C, Poganik JR, Justice J, Belsky DW, Higgins-Chen A, et al. Biomarkers of Aging for the Identification and Evaluation of Longevity Interventions. Cell. (2023) 186:3758-75. doi: 10.1016/j.cell.2023.08.003.
